# LncRNAs regulating stemness in aging

**DOI:** 10.1111/acel.12870

**Published:** 2018-11-20

**Authors:** António Sousa‐Franco, Kenny Rebelo, Simão Teixeira da Rocha, Bruno Bernardes de Jesus

**Affiliations:** ^1^ Instituto de Medicina Molecular Faculdade de Medicina da Universidade de Lisboa Lisboa Portugal; ^2^ Department of Medical Sciences and Institute of Biomedicine—iBiMED University of Aveiro Aveiro Portugal

**Keywords:** aging, epigenetics, long non‐coding RNAs (lncRNAs), stem cells

## Abstract

One of the most outstanding observations from next‐generation sequencing approaches was that only 1.5% of our genes code for proteins. The biggest part is transcribed but give rise to different families of RNAs without coding potential. The functional relevance of these abundant transcripts remains far from elucidated. Among them are the long non‐coding RNAs (lncRNAs), a relatively large and heterogeneous group of RNAs shown to be highly tissue‐specific, indicating a prominent role in processes controlling cellular identity. In particular, lncRNAs have been linked to both stemness properties and detrimental pathways regulating the aging process, being novel players in the intricate network guiding tissue homeostasis. Here, we summarize the up‐to‐date information on the role of lncRNAs that affect stemness and hence impact upon aging, highlighting the likelihood that lncRNAs may represent an unexploited reservoir of potential therapeutic targets for reprogramming applications and aging‐related diseases.

## 1 INTRODUCTION AND CONTEXT

The rapid progression of next‐generation sequencing (NGS) has produced an enormous amount of descriptive data on the expression profiles of several coding and non‐coding transcripts (Carninci et al., 2005). Different consortiums, namely the ENCODE project, mapped expression data in a variety of cell types and conditions including stem, progenitor, and somatic cells (Bernstein et al., 2012). One of the first surprises came with the observation that the amount of coding genes was lower than initially expected and was paralleled with an exponential identification of RNA species lacking coding potential. Additionally, high interspecies variance at the non‐coding level was encountered, suggesting a role for non‐coding transcription in determining species identity (Mattick & Makunin, 2006). Furthermore, it has been demonstrated that non‐coding RNAs present much higher tissue specificity than protein‐coding genes, highlighting their importance for tissue‐specific function/identity (Cabili et al., 2011). Non‐coding RNAs play important regulatory roles in modulating transcriptionally and post‐transcriptionally the coding transcriptome (Angrand, Vennin, Bourhis, & Adriaenssens, 2015; Mattick & Makunin, 2006), which starts to be unveiled in pathological conditions such as cancer. However, how the non‐coding transcriptome diverges from cellular stemness to tissue commitment and aging, and they impact on those processes, remains elusive.

**Figure 1:**
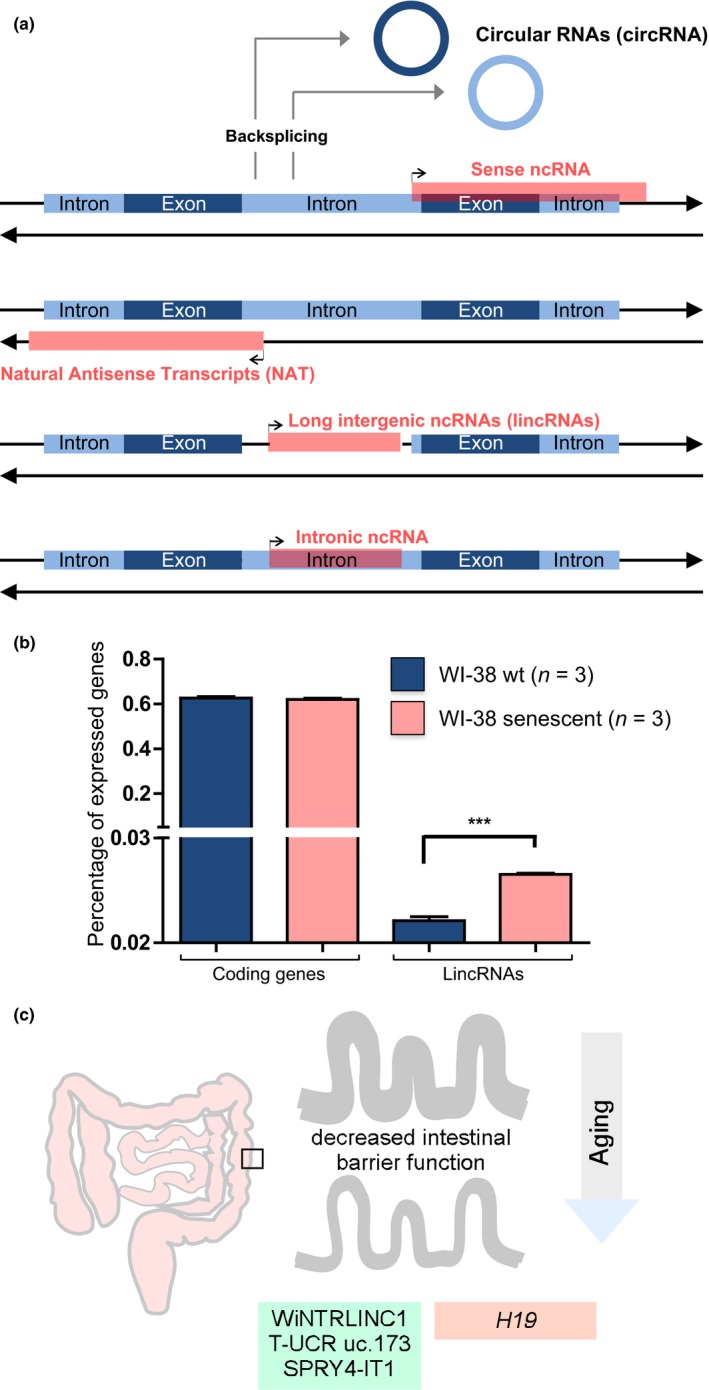
(a) Classification of lncRNAs. lncRNAs can adopt different classifications depending on its localization. LncRNAs can be segments of protein‐coding transcripts or being transcribed from the opposite strand (natural antisense transcripts—NATs). Antisense lncRNAs could be complementary to the antisense strand of protein‐coding or non‐coding genes. lncRNAs could emerge from intergenic regions (lincRNAs) or from introns of coding genes. Protein‐coding exons shown in dark blue and introns in light blue; lncRNAs shown in red. Additionally, IncRNAs can adopt a circular structure of covalently closed loops (circRNAs; Nigro et al., 1991; Rong et al., 2017). circRNAs could be classified into several subtypes depending on their positioning relatively to the parental linear transcript or from the integration of 1 or multiple introns and/or exons (Qu et al., 2017; Westholm et al., 2014; Zhang, Wang, et al., 2014; Zhang, Zhang, et al., 2013). (b) Expression profiles of different RNA species during senescence of human skin fibroblasts. Previously released RNA‐seq data from human *wt* and senescent WI‐38 human cells (Chen et al., 2012; Marthandan et al., 2015) were analyzed with ISAT2(v2.1.0)/Stringtie(v1.3.3b; Kim et al., 2015; Pertea, Kim, Pertea, Leek, & Salzberg, 2016) using Ensembl *Homo sapiens* GRCh37.74 release as template for quantification. FPKM values for each transcript were converted to log2. The threshold value chosen to identify expressed protein‐coding genes was determined as previously described. (Hart, Komori, LaMere, Podshivalova, & Salomon, 2013) and for antisense and lincRNAs when FPKM > 1. Plotted values correspond to the percentage of expressed genes. Two‐sided Student’s *t* test was used for statistical analysis (***p < 0.001). (c) lncRNAs involved in gut homeostasis. In mammals, aging is associated with decreased intestinal barrier function. Differentially expressed lncRNAs may be positively involved in the response of the gut epithelium to the aging stress or, on the other hand, exacerbate the impact of aging on gut function (related to Table 1)

The non‐coding transcriptome encloses a variety of RNA species, spanning from small non‐coding RNAs, including microRNAs (miRNAs), Piwi RNAs (piRNAs), and small nucleolar RNAs (snoRNAs) to long non‐coding RNAs (lncRNAs), that are >200 bp long, but could be as large as several kilobases and be subdivided into different categories. LncRNAs are transcribed majorly by Pol II and Pol I RNA polymerases and are present throughout the genome, either as antisense of coding genes (natural antisense transcripts—NATs), pseudogenes, or intergenic (long intergenic non‐coding RNAs‐lincRNAs), additionally they could be bidirectional, arise from trans‐splicing or adopt different structural forms which increase their stability (Figure 1a) Several lncRNAs have been implicated in gene‐regulatory networks performing roles such as chromosome dosage compensation, genomic imprinting, epigenetic regulation, cell cycle control, splicing, and cell differentiation (Mercer, Dinger, & Mattick, 2009; Rinn & Chang, 2012). Mutant mouse strains for different lncRNAs (*Fendrr*,* Peril*,* Mdgt*,* Brn1b,* or *Pint*) revealed phenotypes ranging from growth defects to abnormalities in the structure of the neocortex (Sauvageau et al., 2013). This study and others were a proof of concept that, similarly to coding genes, lncRNAs might play critical roles in vivo (Li & Chang, 2014). However, considering that lncRNAs account for 10% in mice and 24% in humans of all RNA transcripts (Atianand & Fitzgerald, 2014), the number of lncRNAs with an assigned function is still limited.

Aging is a biological process characterized by a cascade of biochemical changes which result, ultimately, in an observable functional decay (Lopez‐Otin, Blasco, Partridge, Serrano, & Kroemer, 2013) caused by the accumulation of senescent cells which are cells with an irreversible proliferative arrest (de Jesus & Blasco, 2012). Manipulation of the number of senescent cells impacts in the aging progression, demonstrating the feasibility of antisenescence therapies for age‐associated syndromes (Baker et al., 2016, 2011 ; Campisi & d'Adda di Fagagna, 2007; de Jesus & Blasco, 2012; Gil & Withers, 2016; Itahana, Campisi, & Dimri, 2007). Similarly to the lncRNA footprint existing and correlating with the complexity of different tissues, the percentage of expressed lncRNA genes during senescence of primary human fibroblasts better reflects the different cell identities, when compared with the expression of coding genes (Figure 1b). Previously, a “footprint” of several senescence‐associated lncRNAs (SAL‐RNAs) has been identified (Abdelmohsen et al., 2013), highlighting a link between lncRNAs and aging. Furthermore, targeting of selected lncRNAs (e.g., SAL‐RNA1—XLOC_023166) was found to actually delay senescence, suggesting a direct role for lncRNAs in the acquisition and/or maintenance of senescence features.

Additionally to their sequence‐dependent role, lncRNAs may adopt different structures with the same sequence, leading to different biological properties. One example are the circular RNAs. One curious example is the antisense transcript coexisting in the *INK4a‐ARF* locus (a tumor suppressor associated to stemness, aging, and cancer; Li et al., 2009) named ANRIL (Aguilo, Zhou, & Walsh, 2011; Holdt et al., 2016). ANRIL could adopt a linear and/or circular form. ANRIL has 19 exons (Burd et al., 2010; Pasmant et al., 2007) resulting in several alternatively spliced transcripts (Folkersen et al., 2009). Interpreting the biological function of ANRIL has become increasingly complicated. ANRIL has been shown, for instance, to regulate neighbor tumor suppressor genes in *cis* by epigenetic mechanisms (Lee, 2012) and to correlate with atherosclerotic vascular disease risk through novel circular isoforms (cANRIL; Burd et al., 2010). These studies correlate ANRIL structure and function, guiding to the possibility that manipulation of specific ANRIL structure may alter specific cellular processes such as aging. LncRNAs have also been shown to actively participate directly or indirectly on other age‐related pathways such as nutrient sensing (Dang, 2014; Meng et al., 2007; Mourtada‐Maarabouni, Pickard, Hedge, Farzaneh, & Williams, 2009; Wang, Pang, et al., 2014; Zhang, Zhu, et al., 2013), telomere dynamics (Azzalin & Lingner, 2008; Azzalin, Reichenbach, Khoriauli, Giulotto, & Lingner, 2007; Cao et al., 2009; Cusanelli & Chartrand, 2014; Montero, Lopez de Silanes, Grana, & Blasco, 2016; Schoeftner & Blasco, 2008, 2009a, 2009b), and p53‐associated and epigenetically regulated senescence (Bracken et al., 2007; Dietrich et al., 2007; Gil, Bernard, Martinez, & Beach, 2004; Jacobs, Kieboom, Marino, DePinho, & Lohuizen, 1999; Marin‐Bejar et al., 2013; Puvvula et al., 2014). The role of lncRNAs on these pathways has been recently addressed by others (Degirmenci & Lei, 2016). In this review, we focus on the role of lncRNAs on different cellular networks regulating stemness in aging and on the impact of aging in cellular reprogramming processes.

## 2 STEMNESS AND AGING

### 2.1 Impact of aging on adult stem cells

Stem cells have the potential to self‐renew and to differentiate into different lineages, being a source of different adult specialized cell types and tissues (Watt & Hogan, 2000). Most adult organs retain a limited regenerative capacity which seems to depend on the stem cells reserves (which maintain self‐renewal and pluripotency potential after mobilization signals; Bianco & Robey, 2001; Korbling & Estrov, 2003). Although stem cells have specialized characteristics which protect them from external insults, aging impacts on stem cell homeostasis, resulting in halted stem cell renewal and proliferation (Ermolaeva, Neri, Ori, & Rudolph, 2018; Goodell & Rando, 2015). Stem cells experienced aging‐dependent accumulation of DNA damage and telomere shortening (Flores & Blasco, 2010; Flores et al., 2008), directly impacting on stem cell function and ultimately on lifespan (Ruzankina et al., 2007; Vilas et al., 2018). Interestingly, at least some of the phenotypes of stem cell aging may be partially delayed. An example is the anti‐aging effects of caloric restriction (Mazzoccoli, Tevy, Borghesan, Delle Vergini, & Vinciguerra, 2014). Caloric restriction was shown to prolong the capacity of stem cells to self‐renew, proliferate, differentiate, and replace cells in several adult tissues. Whether lncRNAs may be acting directly or indirectly on stem cell homeostasis and be potential novel targets for stem cell resistance to aging‐induced processes has been recently come to stage (Chen, Zhu, et al., 2017; Bernardes de Jesus et al., 2018; Li et al., 2017; Ramos et al., 2013).

Adult stem cells are a rare population of undifferentiated cells capable of self‐renewal and to differentiate into lineage‐specific tissues usually within the niche they reside (Dulak, Szade, Szade, Nowak, & Jozkowicz, 2015). Adult stem cells replace damaged cells due to tissue turnover or injury. High turnover organs are known to be populated by adult stem cells, although it is believed several adult tissues retain populations of adult stem cells even in the absence of detectable proliferation (Dulak et al., 2015). Well‐characterized examples of high turnover tissues are the intestine, blood, or muscle. Here, adult stem cells play crucial roles in tissue homeostasis (Wagers & Weissman, 2004). During the lifespan of a person, adult stem cells also age, being this concomitant with a decline in their properties (Ahmed, Sheng, Wasnik, Baylink, & Lau, 2017). Aging affects mostly, but not only, high turnover tissues such as the bone marrow‐derived mesenchymal stem cells and subsequently the hematopoietic stem cells (HSCs), the skeletal muscle, or the intestine. Whether lncRNAs play a role in adult stem cell aging remains to be fully demonstrated. Hereafter, we will describe the role of known lncRNAs in adult stem cells and their potential correlation with the aging process in distinctive tissues.

#### 2.1.1 lncRNAs in adult skeletal muscle stem cells

Adult skeletal muscle retains partial capacity to regenerate (Ahmed et al., 2017; Brack & Munoz‐Canoves, 2016; Garcia‐Prat, Sousa‐Victor, & Munoz‐Canoves, 2013), thanks to the existence of adult muscle stem cells also known as satellite cells. The impaired capacity of skeletal muscle to regenerate, in particular after injury during aging, may be due to the decline of tissue function and muscle stem cells properties. Indeed during aging, satellite cells display a delayed response to activating stimuli resulting in a reduced proliferative response (Brack et al., 2007; Conboy, Conboy, Smythe, & Rando, 2003; Garcia‐Prat et al., 2013; Schultz & Lipton, 1982; Taylor‐Jones et al., 2002). Several lncRNAs have been described in the processes regulating muscle differentiation and regeneration (Hagan et al., 2017). LncRNAs involved in myogenesis include *Malat1*,* linc‐RAM*,* MUNC*,* lnc‐mg*, and *linc‐31*. Using both in vitro and in vivo assays, Chen et al. demonstrate that *Malat1* regulates gene expression during myogenic differentiation (Chen, He, et al., 2017). The molecular mechanism proposes that in the proliferating myoblasts, *Malat1* is highly abundant and leads to trimethylation of the histone 3 lysine 9 (H3K9me3) and subsequent repression of the target gene expression by recruiting Suv39h1 to MyoD‐binding loci. During differentiation, *Malat1* is degraded, thus destabilizing the repressive complex and leading to target gene activation. Together, Chen et al. identified a regulatory axis in myogenesis controlled by *Malat1*, showing an inhibitory role for *Malat1* during myogenic differentiation. *Linc‐RAM* is involved in the differentiation stage of myogenesis by regulating the transcription of *MyoG* (Yu et al., 2017). The lncRNA *MUNC* targets RNAs such as *myogenin* and *Myh3* involved in myogenic differentiation (Mueller et al., 2015). *Lnc‐mg* is specifically enriched in skeletal muscle and is essential for muscle cell differentiation and skeletal muscle development (Zhu et al., 2017). Lastly, Dimartino et al show that *lnc‐31*, a lncRNA required for myoblast proliferation, stabilizes the YB‐1 factor, allowing its positive effect on *Rock1* mRNA translation (Dimartino et al., 2018; see Table 1). Other muscle‐specific lncRNAs include the *LincMD1*, which controls muscle differentiation by acting as a competitive endogenous RNA (ceRNA) of miR‐133 and miR‐135 regulating the expression of *MAML1* and *MEF2C* (Cesana et al., 2011). Overexpression of *linc‐MD1* correlates with the anticipation of the muscle differentiation program. Although they proved involvement in muscle regeneration programs, the correlation of muscle lncRNAs with the aging process is still missing. Recently, a novel lncRNA *(Chronos)* has been identified in aged muscle (Neppl, Wu, & Walsh, 2017). *Chronos* is regulating the process leading to the gradual loss of muscle mass occurring with advancing age. *Chronos* is positively regulated with age. Inhibition of *Chronos* induces hypertrophy of the muscle through the modulation of *Bmp7* signaling (Neppl et al., 2017).

**Table 1:** LncRNAs regulating stem cells in adult organs

Names	Mechanism	References
Adult skeletal muscle stem cells		
MALAT1	MyoD suppression through Suv39h1/HP1β/HDAC‐1	Chen, He, et al. (2017))
linc‐RAM	Enhance MyoG transcription through MyoD‐Baf60c‐Brg1	Yu et al. (2017)
MUNC	Increase myogenic‐related mRNAs	Mueller et al. (2015)
lnc‐mg	Myogenic signaling (IGF2)	Zhu et al. (2017)
Linc‐31	Required for myoblast proliferation	Dimartino et al. (2018)
linc‐MD1	Controls muscle differentiation (ceRNA)	Cesana et al. (2011)
Chronos	Induces hypertrophy of the muscle through the modulation of Bmp7	Neppl et al. (2017)
Adult hematopoietic stem cells		
lncHSC‐1	Regulate HSC differentiation via cell cycle and chromatin regulators	Luo et al. (2015)
lncHSC‐2	Regulate HSC differentiation via cell cycle and chromatin regulators	Luo et al. (2015)
Spehd	Silencing lead to defective multilineage differentiation	Delás et al. (2018)
Gut		
WiNTRLINC1	Controls intestinal stem cell fate through ASCL2	Giakountis et al. (2016)
T‐UCR uc.173	Stimulates growth of the small intestinal mucosa	Xiao et al. (2018)
H19	Disrupts the gut epithelium by degradation of ZO‐1 and E‐cad mRNAs	Zou et al. (2016)
SPRY4‐IT1	Controls the expression of several tight junctions’ proteins	Scherr et al. (2007)

#### 2.1.2 LncRNAs and HSC

Hematopoietic stem cells (HSCs) are specialized blood‐forming stem cells (Birbrair & Frenette, 2016) which maintain self‐renewal during an entire lifespan. HSCs also produce immune cells assuring immune protection. HSCs activity is regulated by cell‐intrinsic and cell‐extrinsic mechanisms. Aging affects this regulatory network, leading to a decrease in number of HSC characterized by impaired function (Pietras, Warr, & Passegue, 2011). Luo and colleagues compared lncRNA expression between different HSC ages (aged HSCs exhibit a repopulation defect) and between WT and DNA methylation‐deficient Dnmt3a KO HSCs (Dnmt3a^−/−^ HSCs exhibit defective differentiation) (Challen et al., 2011). They focused on two lncRNAs, *LncHSC‐1* and *LncHSC‐2*, which are highly expressed in WT HSC, but absent in *Dnmt3a* KO HSCs. Additionally, they also identified a small subset of lncRNAs (29 out of 159) with altered expression between 4mo and 24mo HSCs. Surprisingly, the lncRNAs whose expression was changed with aging were not characterized (Luo et al., 2015). Whether the aging‐related lncRNAs may play a similar role in increasing colony formation in the context of aging is currently unknown (Figure 1c). Recently, Delás and colleagues characterized a subset of mouse lncRNAs with potentially relevant expression during hematopoietic differentiation. Among the candidates was identified one lncRNA, *Spehd*, which silencing lead to myeloid progenitors deficiency in their oxidative phosphorylation pathway (Delás et al., 2018). With the increasing interest in lncRNAs and the advent of novel technologies, we believe the future will bring major findings on the biology of lncRNAs on HSC dynamics during aging.

#### 2.1.3 Gut

The gut epithelium is a self‐renewing tissue dependent on an intricate process including mobilization, proliferation and differentiation of basal stem cells. The fast division and mobilization of novel cells need to be counterbalanced by a well‐regulated apoptotic process (Wang & Xiao, 2017). This balance is regulated by internal and external cues. Disruption of the gut epithelial may occur in patients with serious diseases, leading to the passage of toxic substances to the blood. Similarly to other genotoxic signals, aging leads to a severe change in the gut homeostasis (Wang & Xiao, 2017). In *Drosophila*, aging results in an increased number and proliferation of dysfunctional stem cells (Moorefield et al., 2017; Tran & Greenwood‐Van Meerveld, 2013). In mammals, aging is associated with decreased intestinal barrier function (Tran & Greenwood‐Van Meerveld, 2013) and impaired nutrient absorption (Holt, 2007). Mouse models of accelerated aging indicate phenotypic changes in the gut epithelium including faulty regeneration, deregulation of stem cell division capacity (Fox, Magness, Kujoth, Prolla, & Maeda, 2012), and altered canonical Wnt signaling (Liu & Rando, 2011), a pathway involved in stem cell maintenance and mobilization. Giakountis et al. (2016) described an lncRNA named *WiNTRLINC1* which positively regulates the expression of *ASCL2*, a transcription factor that controls intestinal stem cell fate. *WiNTRLINC1* and *ASCL2* form a feed‐forward regulatory loop that controls stem cell‐related gene expression. This regulatory circuitry was shown to participate in colorectal cancer progression. Whether it may have a role in aging is still unknown. Other classes of RNAs involved in gut homeostasis are the lncRNAs transcribed from ultra‐conserved regions (T‐UCRs). Xiao and colleagues described the expression patterns of T‐UCRs in the intestinal epithelium (Xiao et al., 2018). T‐UCRs exhibited distinct dynamics after food starvation. Here, T‐UCR uc.173 stimulated growth of the small intestinal mucosa. Due to the conservation observed by this class of transcripts, these findings may provide a venue for therapeutic strategies stimulating the regeneration of the intestinal mucosa such as during aging (Xiao et al., 2018). Other lncRNAs participating in the gut biology are the lncRNA *H19* and the lncRNA *SPRY4‐IT1*. *H19* is a conserved lncRNA transcribed from the imprinted *H19/Igf2* gene cluster. *H19* is highly expressed during embryogenesis, but its levels decrease during aging (Fu et al., 2008). *H19* is a molecular sponge or bind to different miRNAs (Kallen et al., 2013). *H19* abundance disrupts the gut epithelial function probably by enhancing the degradation and repressing the translation of zonula occludens protein 1 (ZO‐1) and E‐cadherin mRNAs (Zou et al., 2016), two proteins with functional roles in forming and regulating the epithelial barrier (Bhatt, Rizvi, Batta, Kataria, & Jamora, 2013; Furuse, Izumi, Oda, Higashi, & Iwamoto, 2014; Tian et al., 2011; Zou et al., 2016). Other studies further demonstrate that ectopically expression of *H19* induces the levels of several miRNAs (miR‐675‐3p or miR‐675‐5p) in intestinal epithelial cells (IECs) (Dey, Pfeifer, & Dutta, 2014). Epithelial barrier dysfunction may be a response to increased levels of those miRNAs. Similarly to the scenario observed in cancer, loss of imprinting of the *IGF2‐H19* locus during aging (Fu et al., 2008) may be leading to an abnormal expression of *H19*, and other genes in this locus, leading to a dysfunctional mobilization of gut stem cells (Grammatikakis, Panda, Abdelmohsen, & Gorospe, 2014). Another example is *SPRY4‐IT1*, a lncRNA widely expressed among different human tissues including the intestinal mucosa (Khaitan et al., 2011). *SPRY4‐IT1* enhances the gut epithelial barrier function by increasing tight junctions (Xiao et al., 2016). *SPRY4‐IT1* is highly expressed in gut stem cells. Silencing of *SPRY4‐IT1* inhibits expression of several tight junctions’ proteins disrupting the epithelial barrier function. Lentiviral expression of *SPRY4‐IT1* (Scherr et al., 2007) protects the gut barrier in mice exposed to external stresses. Interestingly, mucosal *SPRY4‐IT1* levels decrease in patients diagnosed with increased gut permeability (IGP) comparing to normal‐mucosal samples from controls (Wang & Xiao, 2017). *SPRY4‐IT1* levels correlate with repressed levels of tight junctions guiding to the potential role for this lncRNA in reverting altered mucosa phenotypes (Wang & Xiao, 2017). Manipulation of these lncRNAs may prove beneficial for age‐dependent gut loss of homeostasis.

## 3 AGING ROADBLOCKS DURING CELLULAR REPROGRAMMING—A ROLE FOR lncRNAs?

Several alternatives in vitro methodologies have been optimized for the reprogramming and/or expansion of embryonic‐like stem cells from adult tissue. In particular, Yamanaka and colleagues found that expression of four transcription factors, namely *Sox2, Klf4, Oct4*, and *c‐Myc*, in adult human and mice skin fibroblasts converts them to a “stem‐like” condition named induced pluripotent stem cells (iPSCs; Takahashi & Yamanaka, 2006; Yamanaka, 2009). The possibility to replace the original retroviral and lentiviral vectors through the use of nonintegrative strategies was tested and is being used since then (Sun, Longaker, & Wu, 2010), and this included non‐coding RNA players. Indeed, soon after the release of the initial iPSC reprogramming protocol, a report revealed that introducing miRNA mimics of embryonic stem cells (ESCs) specific miRNAs enhanced mouse iPSC derivation and replaced the function of *c‐Myc* during reprogramming (Judson, Babiarz, Venere, & Blelloch, 2009). Scrutinizing the differential distribution of the coding and non‐coding transcriptome between stem and differentiated cells may unveil novel targetable reprogramming barriers. Due to the gain of regenerative potential during cellular reprogramming, it has been thought as useful to the aging field (Ocampo, Reddy, & Belmonte, 2016; Soria‐Valles & Lopez‐Otin, 2016). Induced pluripotent cells obtained during cellular reprogramming of aged tissue reset their stress‐ and senescence‐associated epigenetic marks (Lapasset et al., 2011; Liu et al., 2011; Zhang et al., 2011). Erasure of the aging marks is a crucial step during cellular and tissue regeneration strategies.

Aging has been identified as an obstacle in the iPSC reprogramming process. Indeed, reprogramming of aged cells into iPSCs is a very inefficient process, resulting in cells which do not pass the intermediate states and do not fully acquire pluripotency characteristics. Several barriers have been described in aged cells which could account to this limitation. Among the pathways involved, cellular senescence may be one of the key barriers, at least in mice (Banito et al., 2009; Hong et al., 2009; Kawamura et al., 2009; Li et al., 2009; Marion et al., 2009; Tat, Sumer, Pralong, & Verma, 2011; Utikal et al., 2009; Zhao et al., 2008). Senescent cells are characterized by an irreversible cell cycle arrest, higher expression of the *ink4a/ARF* locus, and several changes at the cellular characteristics such as chromatin condensation and secretory phenotypes (Campisi & d'Adda di Fagagna, 2007; de Jesus & Blasco, 2012; Kuilman, Michaloglou, Mooi, & Peeper, 2010). Cellular reprogramming was shown to be strictly dependent on the division capacity of cells (Hanna et al., 2009; Hanna, Saha, & Jaenisch, 2010), being this loss an hallmark of senescence. Another barrier detected during aging that may be affecting the efficiency of cellular reprogramming is changes affecting the mTOR (target of rapamycin) pathway. TOR inhibitors may act by facilitating a mesenchymal‐to‐epithelial transition (MET; Chen et al., 2011), as cells of mesenchymal origin such as adult fibroblast undergo MET during cellular reprogramming (Li et al., 2010; Samavarchi‐Tehrani et al., 2010). Indeed, expression of *Zeb2* (Beltran et al., 2008; Wang, Guo, et al., 2013), an EMT factor, is shown to increase with aging and to be a barrier for cellular reprogramming (Bernardes de Jesus et al., 2018). Downregulation of *Zeb2* in aged/old adult fibroblasts greatly impacts on their reprogramming efficiency (Bernardes de Jesus et al., 2018). The reduced efficiency of reprogramming of aged cells might indicate failure of many cells to fully commit to the stem‐like state. Furthermore, whether iPSCs from aged‐derived cells present the same hallmarks of pluripotency as young‐derived ones has not been systematically analyzed. In this respect, old donor cells have been found to be resistant to the normal demethylation during human reprogramming, resulting in ~5% increase in global methylation levels (Lo Sardo et al., 2017). This points out for the likelihood of iPSCs from older donor cells to accumulate more stochastic epigenetic errors during reprogramming, which might impact on the expression of imprinted lncRNAs and result in iPSCs of reduced pluripotent potential.

### 3.1 LncRNAs as part of the stem cell network

LncRNAs have long been associated with cellular stemness (Loewer et al., 2010) with more than 100 lncRNAs known to bind to pluripotency transcription factors (Sheik Mohamed, Gaughwin, Lim, Robson, & Lipovich, 2010). Several lncRNAs showed direct involvement in the maintenance of pluripotency, regulating directly the levels of transcription factors (TFs), or participating in the reprogramming process (Guttman et al., 2011; Loewer et al., 2010). The synergy between lncRNAs and stemness is further confirmed by the direct association of pluripotency TFs, such as Oct4, Sox2, or Nanog to lncRNAs promoters, suggesting a direct regulation of lncRNAs levels in cell reprogramming and stemness preservation (Loewer et al., 2010). One example is the *lncRNA‐RoR* which was shown to participate in the reprogramming conversion (Wang, Xu, et al., 2013). *lncRoR* works as a miRNA sponge, protecting pluripotency TFs from miRNA targeting. A pluripotency candidate directly regulated by lncRNAs is the oncogene *c‐Myc*. Although it was traditionally associated with cancer (Dang, 2012) and, possibly, a secondary player during somatic cell reprogramming, the presence of *c‐Myc* in the reprogramming cocktail increases the yield of iPSCs. Recently, it has been described that a non‐coding transcript, named *PVT1* lncRNA, present in the vicinity of the *c‐Myc* locus, appears to increase stability of the c‐Myc protein by, protecting c‐Myc protein from phosphorylation‐mediated degradation, maintaining high levels of Myc (Tseng et al., 2014).

Regulation of stem cell differentiation toward committed lineages by lncRNAs is yet poorly characterized. Murine ESCs remain undifferentiated in the presence of leukemia inhibitory factor (LIF), which works through activation of the signal transducer and activator of transcription 3 (STAT3; Cartwright et al., 2005). Recently, it was observed that down‐regulation of *lncDC*, a novel lncRNA expressed in human conventional dendritic cells (DCs; Wang, Xue, et al., 2014), impaired DC differentiation from mouse bone marrow cells, both in vitro and in vivo. These effects were mediated by the activation of the transcription factor STAT3, through direct binding of *lncDC* to STAT3 in the cytoplasm, which promoted STAT3 phosphorylation. These findings are in line with previous studies demonstrating a role for lncRNAs beyond chromatin remodeling. The identification of stem cell‐specific lncRNAs may lead to the characterization of lncRNAs important in stem cell identity and in the identification of novel barriers limiting the reprogramming process in particular of aged cells.

### 3.2 lncRNAs and epigenetic rewiring during reprogramming

Before the discovery of the extensive non‐coding transcription across the genome from high‐throughput studies, lncRNAs were long known to be players in the epigenetic processes of X‐chromosome inactivation (XCI) and genomic imprinting (Lee & Bartolomei, 2013). Genomic imprinting is an epigenetic phenomenon that renders a subset of genes to be mono‐allelically expressed according to their parental origin (Barlow & Bartolomei, 2014). These genes are frequently located in the same genomic regions, commonly known as imprinted clusters, an organization implying a common mechanism of imprinting regulation. Indeed, all imprinted clusters have *cis*‐acting imprinting control regions (ICRs) which are epigenetically differentially marked by DNA CpG methylation in the two parental alleles. Interestingly, most imprinted clusters have at least one lncRNA which is mono‐allelically expressed and regulated by CpG DNA methylation. These lncRNAs can be intergenic or antisense to reciprocally imprinted genes. They are believed to regulate imprinted expression of the neighboring genes through the act of transcription itself or by the recruitment of chromatin‐modifying complexes, as has been referred to the cases of *Airnc*,* Kncq1ot1*, and *Meg3* lncRNAs (Kaneko, Son, Bonasio, Shen, & Reinberg, 2014; Latos et al., 2012; Nagano et al., 2008; Terranova et al., 2008). Such studies paved the way for the investigation of the role of many lncRNAs and their link with the epigenetic machinery namely methylating/demethylating enzymes and chromatin‐modifying complexes for instance (Quinn et al., 2016). Epigenetically related lncRNAs may be involved in the aging process. For example, *Xist* lncRNA is known to become downregulated during senescence in vitro (Abdelmohsen et al., 2013). Recent genome‐wide studies clearly pointed out for an epigenetic clock in both mouse and human tissues based on aging‐related DNA methylation changes (Hannum et al., 2013; Horvath, 2013; Stubbs et al., 2017; Weidner et al., 2014). Indeed, a DNA methylation signature of aging was uncovered and capable of predicting the chronological age and functional decline of a given tissue (Horvath, 2013; Stubbs et al., 2017). Whether such epigenetic changes are a cause or a consequence of the aging process still needs to be uncovered.

During iPSC reprogramming, a massive epigenetic rewiring of the differentiated program into the stem‐like state occurs in a short time window. Aged cells encounter an extra layer of epigenetic rewiring since it requires not only an epigenetic resetting of the donor cell memory, but also of their aging‐specific characteristics (Hochedlinger & Plath, 2009; Mertens et al., 2015). This might explain their decreased efficiency in reprogramming, as elucidated clearly from mouse cells studies (Mahmoudi & Brunet, 2012). For instance, while full reversal of aging‐specific epigenetic features is believed to occur (Mertens et al., 2015), some might persist (Lo Sardo et al., 2017), which might hinder the pluripotency capacity and quality of iPSCs derived from aged donor cells.

Another aspect is that epigenetic‐sensitive loci such as imprinted regions could be deregulated during this process. Indeed, imprinting errors have been documented in both mouse and human iPSCs (Ma et al., 2014; Nazor et al., 2012; Stadtfeld et al., 2010; Sun et al., 2012), giving rise to inappropriate silencing or biallelic expression of imprinted genes, including imprinted lncRNAs. In particular, these errors are recurrent at the *Dlk1‐Dio3* imprinted cluster, where hypermethylation leads to the loss of expression of several imprinted non‐coding transcripts including the *Meg3* and *Meg8* lncRNAs (Ma et al., 2014; Stadtfeld et al., 2010). As a consequence, these iPSCs lose their pluripotency hallmarks. Indeed, *Meg3* ^OFF^ mouse hiPSCs contribute poorly in chimeric mice and fail to generate “all‐iPSC” mice, the most stringent pluripotent test (Carey et al., 2011; Liu et al., 2010; Stadtfeld et al., 2010). Likewise, *MEG3* ^OFF^ human iPSCs fail to differentiate properly down the neuronal lineage (Mo et al., 2015). These results indicate a major role for *Dlk1‐Dio3* imprinting in pluripotency and suggest the involvement of imprinted lncRNAs in determining the full developmental potential of iPSCs. Whether this stochastic epigenetic errors affecting imprinting during the inefficient process of iPSC reprogramming of aged cells is exacerbated and whether they can explain, to some extent, their reduced inability to become iPSCs and the role of imprinted lncRNAs on these processes are interesting areas of research to follow.

### 3.3 MET transition during reprogramming of aged cells

A mesenchymal‐to‐epithelial transition (MET) is the first important decision that cells undergoing reprogramming need to overcome (Sancho‐Martinez & Izpisua Belmonte, 2013), especially if using the favorite mesenchymal‐derived dermal fibroblasts as donor cells (Li et al., 2010). Importantly, forced expression of E‐cadherin (epithelial marker) (Redmer et al., 2011) or downregulation of *Zeb2*, which facilitates MET transition, augmentes the efficiency of reprogramming (Wang, Guo, et al., 2013). Whether MET may be delayed during reprogramming of aged iPSCs and act as an aging barrier for reprogramming has been recently unveiled by us (Bernardes de Jesus et al., 2018). Moreover, we identified a lncRNA, called *Zeb2*‐NAT, a natural antisense transcript of *Zeb2*, as a molecular target to improve reprogramming of aged cells. (Mattick, 2010; Mercer & Mattick, 2013; Zhang, Yang, & Chen, 2014). NATs are a particular group with very interesting characteristics, in particular due to its antisense transcription with potential regulatory role of the sense protein‐coding genes (Beltran et al., 2008; Bernardes de Jesus et al., 2018; Matsui et al., 2008; Wang, Chung, et al., 2014; Zong et al., 2016). This might be a common regulatory module, since according to recent studies, 72% of mice and human genomic loci are transcribed from both sense and antisense strands (Werner, Carlile, & Swan, 2009). *Zeb2*‐NAT overlaps Zeb2 5′UTR region and leads to the retention of its first intron, which harbors an IRES sequence resulting in the functional translation of a Zeb2 protein. Interestingly, *Zeb2* and *Zeb2*‐*NAT* expression seems to correlate with the aging process, being highly expressed in old fibroblasts. Additionally, *Zeb2*‐NAT seems to precede the expression of *Zeb2* RNA in differentiation protocols (Bernardes de Jesus et al., 2018). In particular, it was observed that *Zeb2‐NAT* expression precedes the expression of their antisense coding pair *Zeb2*, guiding to different regulatory networks, and proving the functional involvement of antisense transcription in cellular reprogramming and aging. Overall, antisense transcription could act locally, interfering in the functional levels of the sense transcript, or as regulatory hubs responsible for the dispersion of regulatory signals to neighboring genes (Pelechano & Steinmetz, 2013). Whether both sense and antisense transcription may be expressed in the same cell, or at the same time, remains to be elucidated. The importance of divergent transcription, as observed in sense–antisense transcription pairs, has been recently assessed by Lou and colleagues who elegantly linked divergent RNAs to cell lineage commitment (Luo et al., 2016). Divergent lncRNAs are shown to be relatively abundant, to co‐localize and to be co‐express with developmental and transcription regulator genes and to be associated with epigenetic marks involved in differentiation regulatory networks (Luo et al., 2016). *Zeb2‐NAT* lncRNA is an example of a lncRNA more expressed in aged cells whose modulation of expression can improve iPSC reprogramming from aged cells (Bernardes de Jesus et al., 2018). It is likely that other lncRNAs might exist with similar attributes which might be revealed by highly sensitive transcriptome studies such as the novel native elongating transcript sequencing technology (mNET‐seq), which generates single‐nucleotide resolution (Nojima et al., 2015) or global run‐on sequencing (GRO‐seq) (Core, Waterfall, & Lis, 2008) among other high‐resolution techniques.

## 4 LncRNAs AS ANTI‐AGING THERAPIES

As mentioned before, lncRNAs are emerging as potential targets for anti‐aging therapies. Their non‐coding nature and particularities (such as the conformational complexity, cellular localization, or interactions) need to be taken into consideration in the design of strategies for efficient lncRNA modulation.

Modified oligonucleotides are probably the best characterized and known approach to target lncRNAs. Antisense oligonucleotides have been traditionally used as a research tool to explore function of several lncRNAs in vitro and in vivo. More recently, novel oligonucleotides harboring RNA or DNA recognition and cleavage domains have shown up as potential novel strategies with an increased specificity and stability to target lncRNAs independently of their cellular compartmentalization (Bhartiya et al., 2012; Jadhav, Scaria, & Maiti, 2009; Lennox & Behlke, 2016; Suryawanshi et al., 2012), in particular when including base modifications such as locked nucleic acids (LNA). Regarding in vivo strategies, nowadays, there are still issues at the level of delivery and targeting due to the fact that different oligonucleotides work in a cell and tissue‐specific manner. Moreover, the route of delivery is sometimes inefficient and could lead to off‐targets. Nevertheless, designed catalytic oligonucleotides harboring base modifications for stability and specificity against lncRNAs are still one of the best strategies to reach satisfactory down‐regulated levels of mature lncRNAs avoiding genetic modifications. Examples include the strategy employed to target Angelman syndrome in mice (Meng et al., 2015). Angelman syndrome is caused by maternal deficiency of *UBE3A*, with the paternal copy of *UBE3A* being silenced by a lncRNA named *UBE3A‐ATS* (Tan & Bird, 2016). Targeting of the mouse *Ube3a‐ATS* with antisense oligonucleotides (ASOs) ameliorated some cognitive deficits associated with the disease (Meng et al., 2015). Whether the same strategy could be used in humans is still unknown. Another example is *SAMMSON*, a lncRNA linked to melanogenesis. Targeting *SAMMSON* through intravenous delivery of ASO in a human xenograft model significantly reduced tumor growth and cell proliferation (Leucci et al., 2016; Matsui & Corey, 2017). Additionally, modified antisense oligonucleotides have been used to effectively treat human conditions such as hypercholesterolemia and inflammatory bowel disease (Marafini et al., 2015; Toth, 2013). The use of modified antisense oligonucleotides in both neuromuscular and neurodegenerative diseases with a monogenic cause has recently advanced to clinical trials (e.g., Duchene muscular dystrophy; Koo & Wood, 2013; Wilton & Fletcher, 2005).

### 4.1 Future directions

Rapid advances in genome sequencing have placed long non‐coding transcripts as a major player in gene regulation. In this review, we placed the current knowledge on the potential roles of lncRNAs in stemness related to aging. On one hand, we discuss functional roles of lncRNAs in stem cell pools during aging and, secondly, their impact on cellular reprogramming of aged cells. We believe that in the near future, functional tests will undoubtedly uncover anti‐aging therapeutic approaches relying on targeting of lncRNAs. We expect many surprises to come, where a complex trait such as aging could be seen at the light of the non‐coding transcriptome.

## CONFLICT OF INTEREST

None declared.
